# Surgical Complications of Typhoid Fever1Read before the Richmond, Va., Academy of Medicine and Surgery, July 11, 1899.

**Published:** 1899-09

**Authors:** Hugh M. Taylor

**Affiliations:** Richmond, Va., Professor of practice of surgery, University College of Medicine; surgeon to Virginia Hospital, etc.


### SURGICAL COMPLICATIONS OF TYPHOID FEVER.[1]

By HUGH M. TAYLOR, M. D., Richmond, Va.,

Professor of practice of surgery, University College of Medicine; surgeon to Virginia Hospital, etc.

[From the *Richmond Journal of Practice*, July, 1899.]

WHAT hope does surgery offer patients seemingly or really doomed by the occurrence of typhoid perforation? It is claimed by Murchison that 90 per cent. to 95 per cent. will die.

1. Read before the Richmond, Va., Academy of Medicine and Surgery, July 11, 1899.

For our part, we are forced to think that a majority of the small number supposed to have recovered spontaneously were cases of mistaken diagnosis,—cases of local peritonitis without perforation. Granting, for the sake of comparison only, that Murchison's claims are correct, and that from 5 per cent. to 10 per cent. of suspected perforations will recover spontaneously, a knowledge of the triumphs of operative surgery in this field leaves no doubt as to its efficiency.

Dr. Keen[2] reduces the authenticated cases operated upon to 60, with 13 recoveries. Since the publication of the above statistics, I have had one case to recover after operation and one to die. This last mentioned case occurred only a few days ago. The young man had been sick with a typical enteric fever for six weeks. For the past week his case had been complicated by retention of urine, induced by a specific urethritis contracted just before he was taken sick with the fever. For a week it was necessary to catheterise him at regular intervals. At 10 a. m. he was doing well—pulse, 108; temperature, 104; no pain; no abdominal distension; morale good. At 10.30 he had a movement from his bowels and passed his water for the first time in a week. Immediately he complained of a sharp abdominal pain diffused over the whole abdomen. I saw him two hours later; an anxious expression, thoracic breathing and clammy skin were noticeable; his abdomen was flat, but rigid; his pulse 130, and temperature 105, and there was absolute abolition of peristalsis. This last-mentioned symptom was carefully looked for by placing first the ear and then a phonendoscope over every part of the abdomen.

The sudden onset of pain, rigid abdominal muscles, thoracic breathing, abolished peristalsis and increased pulse rate were the evidences which warranted me in diagnosticating a perforation, and in ordering immediate preparation for celiotomy. There was no appreciable shock; no abdominal distension; no lessened area of hepatic dulness, and no reduction of temperature. In two hours from the time I saw him, and four hours from the onset of symptoms, I made a median section. On incising the peritoneum, a quantity of bile-colored serum and fluid feces escaped, but no gas. On delivering the cecum and last part of the ileum, two large perforations were found within about twelve inches of the ileo-cecal junction. The perforations were about four inches apart, both involved the free margin of the bowel, and were both ugly-looking lesions. The largest was almost as large as a silver quarter and the smaller as large as

2. Keen, Surgery of Typhoid Fever, p. 227.

a dime. For some distance around the larger the tissues were thickened and indurated to such an extent that a resection was seriously considered. Both lesions were, however, closed by deep and superficial sutures. Prolonged irrigation, washing and wiping of intestines and cavity, was practised. (Even in this short time—four hours—membranous flakes were deposited around and near the bowel perforations.) Gauze drainage was freely used and the abdomen closed.

Although not more than fifty minutes were consumed in the operation, the patient reacted badly, and at least two quarts of hot saline were administered under the skin and hot coffee and whiskey by the rectum, together with morphine, strychnia, digitalin, camphor, etc., hypodermatically. During this condition of shock he sweated profusely. Six hours after the operation the house surgeon reported that he had used the catheter, but had only found a few teaspoonfuls of urine in the bladder. This surprised me, in view of the quantity of hot saline left in the peritoneal cavity and the quantity administered under the skin. I instructed that the catheter be used every three hours, and this was done until his death, which occurred nine hours after the operation. In that time not more than a tablespoonful of urine was secreted. As far as I can tell, this patient died from suppression of urine, probably traumatic in origin, and aggravated possibly by acute gonorrheal infection. A post mortem found the peritoneum dry and no increased evidences of peritonitis. The result of this case was a great disappointment, as operative intervention was undertaken within four hours from the first onset of the symptoms, and I counted myself as being exceptionally fortunate in getting into the abdomen early, and equally so in completing the operation in a reasonably short time.

Seven months ago, I met with a case of perforating typhoid ulcer equally as interesting, and more so, in that the diagnosis was made under more obscure manifestations, and the operative intervention was successful. This case has been reported in several journals as a part of a clinical lecture. According to Dr. W. W. Keen's statistics, this success made 61 operations, with 14 recoveries. I am sorry since my experience within the past few days, the table will have to read 62 operations and 14 recoveries.

A little boy had been sick with a typical typhoid fever for six weeks, had been convalescent for ten days, again had fever for two weeks, and again was free from fever for several days. He was reported to have passed a comfortable night, but awoke early complaining of some pain in his abdomen, and had an action. His mother, thinking he needed it, gave him a teaspoonful of syrup of figs. This was, however, promptly vomited, and several times during the next few hours spells of vomiting occurred. One more attack of sharp pain, which lasted only a few minutes, was experienced, and after that time the child insisted that he was all right and had no pain.

Such was the report given on my visit at 11 o'clock—four hours after the first attack of pain—and, I, of course, recognised suspicious symptoms of intestinal perforation. The child, however, did not look sick enough at that time to justify such a suspicion. He expressed himself as being without pain, and his untroubled countenance confirmed this assertion. There were no evidences of shock, and those who saw him at the onset of the sharp attack could not say that even then he presented any of the symptoms of shock. His pulse was now 115, and his sublingual temperature 101 degrees F. Respiration was not noticeably increased, and his morale was exceptionally good. There was, however, some appreciable rigidity of the abdominal muscles.

An absence of fever for several days, the sudden onset of pain and vomiting, a recurrence of fever and rapid pulse, plus the abdominal rigidity and abolition of peristalsis, was the group of symptoms which made me fear perforation. *Per contra*, was the short, sharp attack of pain and peristalsis, incident to having an action. Was the vomiting due to acute indigestion and the dose of syrup of figs? Was the fever and increased pulse rate a product of ptomaine production and absorption within the intact intestinal tract? Was the muscular rigidity voluntary contraction incident to the fear of pain, or from real pain due to an irritated peritoneum? It is common experience in typhoid fever to have pain without perforation; tympanites is the rule, not the exception, while tenderness on pressure, notably in the ileo-cecal region, is commonly experienced. Time and again I have viewed with anxiety just as typical symptoms of perforation as these mentioned, in which the results showed no perforation to have occurred. I am dwelling so minutely on the symptoms manifested to impress the fact that perforation may coexist with very minor early manifestations, or, in fact, as has been observed by others, with no symptoms at all, and to impress the idea that what is needed in this phase of abdominal surgery is an improved diagnosis, rather than an improved operative technique.

A second visit two hours later—eight hours from the beginning of the acute symptoms—did not lessen my apprehension as to the serious nature of the case. There was no noticeable change, except that his pulse had increased to 125; his rectal temperature had fallen to 100.5 degrees. Twelve hours from the onset of the alarming symptoms there were more marked symptoms of serious intraäbdominal trouble, but still classical symptoms were not conclusive. The face of the child was not expressive of impending danger, its respiration was not disturbed, hepatic dulness was not obliterated, vomiting was not at all frequent, pain and restlessness were not significant.

To offset these favorable indications, his abdomen was still to some extent rigid, and what was especially ominous, as it is in all serious intraäbdominal infection, his pulse was now 140, while his rectal temperature was only 100.5 degrees. The increasing pulse rate and decreasing temperature suggesting ptomaine absorption from sepsis and shock and reduction of temperature incident to shock of sepsis, a confirmatory incision was agreed upon. I incised over the ileo-cecal region, because of the known fact that a large majority of typhoid perforations are found in the ileum within eighteen inches of the ileo-cecal junction.

On incising the peritoneum a quantity of sero-purulent fluid escaped from the peritoneal cavity, but no gas. The cecum was quickly delivered, and not more than twelve inches from the small bowel was examined before the punched out pencil-sized hole in the ileum was discovered. There was but little, if any, appreciable inflammatory change about the intestinal lesion. In fact, it looked as if a cobbler's punch had been driven into a healthy bowel. To close the opening with deep mattress and Lembert sutures was the work of a few minutes. The technique incident to wiping the bowels of deposits, irrigating cavity, applying drainage, and completing the operation in all of its details occupied only thirty minutes. This last fact is mentioned to impress the idea that the technique of dealing with typhoid perforations may in some instances be very simple and quickly completed. The ultimate outcome in this case was an uneventful recovery.

It may look like desperate surgery to subject to celiotomy the cadaverous looking patient ill with typhoid fever for weeks, with the added prospect of prolonged anesthesia and the like. Granting that it is formidable surgery, it is inevitable death without it, and it is criminal practice to withhold a means that has saved 14 cases in 62 operations in the face of an alternative that can offer no better result than a mortality of 100 per cent.

*Colostomy During Typhoid Fever—Recovery.*—A middle aged woman had been sick with enteric fever for four weeks. The case had been typical in all respects, except that constipation had been marked—so much so that high enemas medicated with ox-gall, glycerine, turpentine, peppermint water, etc., had to be used. This treatment had the effect of bringing into the lower bowel great masses of partially inspissated fecal matter, which had to be removed by the nurse with her finger in the vagina and rectum. There had been a great deal of tympanites, pain and hemorrhage and perforation was anxiously watched for. On Saturday evening her temperature was 103½ degrees, pulse 108; general condition satisfactory. One profuse intestinal hemorrhage that night must have been fatal, but for the prompt use of subcutaneous saline, strychnia, etc., given by the nurse. The shock incident to this hemorrhage brought the temperature down to normal and ran the pulse up to 150. The depression continued for twenty-four hours. At the end of that time the patient's abdomen began to be markedly swollen, and high enemas were ineffectual either in inducing a passage of gas or fecal matter. In spite of all resources for her relief, the distension became so great and interfered to such an extent with respiration that death seemed a question of only a few hours. The distension was within the intestines and evidently mechanical, as we could notice through the thin abdominal walls coils of distended bowels, and there was exaggerated peristalsis. As a last resort, an enterostomy or colostomy was decided to be imperative, hoping thereby to empty the bowels, relieve the pressure, and tide the patient over the crisis.

An incision in the right inguinal region was made, the first knuckle of distended bowel presenting was delivered and fastened by safety pins in the incision. The bowel caught proved to be a loop of the ascending colon, and was securely fixed by two pins, passed transversely, so as to include lips of wound and bowel. No sutures were used, but gauze strips were packed around the protruded bowel to prevent leaking into the peritoneal cavity. The bowel was then incised transversely to its long axis, and a drainage-tube (large size) introduced, with the bowel opening on the stretch, and secured by a safety pin, including bowel and tube. The technique was so simple, and so quickly done, that no shock, either from the general anesthesia or operation, was appreciable. As is usual in such cases, there was not the great outpour of gas and fecal matter one would expect; but, by flushing the bowel with saline, a free discharge of gas and fluid feces occurred, with almost immediate relief to the patient. Frequent lavage of the bowel also had the effect of bringing the temperature down from 104 degrees to 101 degrees. Several days after the colostomy was performed, a segment of bowel, situated above the umbilicus, became enormously distended, and could not be emptied by irrigation through the tube, or by siphonage of the stomach, or by rectal enemata. Finally, this local distension became so great, and pressed so seriously upon the lungs and heart, that another opening in the bowel was contemplated. As the mass was so very tympanitic, and was probably the transverse colon, I concluded to pass a small aspirator needle into it. This I did, entering the needle obliquely, and I had the satisfaction of hearing the gas rush out, and in seeing the mass collapse as if a balloon had been punctured.

From this time on the case pursued an uneventful course. The obstruction, which was either a volvulus or fecal impaction at the splenic flexure of the sigmoid, was overcome. The colostomy remains, of course, to be cured by a plastic operation. To my mind, this case represents a life saved by prompt surgical intervention. Not so much by my own limited experience as by the experience of others, I am convinced that we do not value as we should the life-saving possibilities of either an enterostomy or colostomy in desperate cases of ileus. It is a treatment which may tide the patient over an immediate crisis, is easy of execution, and can be done with local or short general anesthesia. It is useful alike in acute obstruction mechanically induced or in that due to septic paresis.

Apart from the relief incident to emptying the distended bowel by irrigating the intestines, we lesson the ptomaine production within the intestines and the systemic infection from this common focus. More than one clinician has impressed the idea that a paretic bowel is no longer a sewer, but is really a reservoir full of infection, and that, in septic peritonitis we have only done a part that is needed when we irrigate and drain the septic peritoneal sac. Complete surgery can only be accomplished by incising and emptying the distended bowel. In the hands of quite a number of practitioners, this practice has been attended with very encouraging results.

